# An immune related signature inhibits the occurrence and development of serous ovarian cancer by affecting the abundance of dendritic cells

**DOI:** 10.1007/s12672-023-00717-z

**Published:** 2023-06-15

**Authors:** Fei Teng, Hong Wei, Xiaoqiu Dong

**Affiliations:** 1 In-Patient Ultrasound Department, The Second Affiliated Hospital of Harbin Medical University, Harbin, China; 2 Ultrasound Department, The Fourth Affiliated Hospital of Harbin Medical University, Harbin, China

**Keywords:** Ovary cancer, Immune, Prediction, Dendritic cell, Tumor inhibition, Signature

## Abstract

Serous ovarian cancer is one of the major causes of cancer related death among women worldwide. The advanced diagnosis worsens the prognosis of patients with serous ovarian cancer. The immune system has an important impact on the progression of ovarian cancer. Herein, we aimed to establish an immune related prognostic signature to assist in the early diagnosis, treatment, and prognostic evaluation of patients with serous ovarian cancer. Multiple public data sets and immune related genes were obtained from various online public databases, and immune related prognostic signatures were developed through differential expression analysis, univariate Cox proportional hazard regression analysis, and the Least Absolute Shrinkage and Selection Operator (LASSO) Cox regression model. The nomogram model, Kaplan–Meier survival curve analysis, receiver operating characteristic (ROC) curve analysis, and decision curve analysis showed that this signature had a good prediction potential. In conclusion, an immune related signature with good prediction efficiency was established through systematic bioinformatics analysis, which may play a tumor inhibition role by affecting the abundance of activated dendritic cells.

**Supplementary Information:**

The online version contains supplementary material available at 10.1007/s12672-023-00717-z.

## Introduction

Ovarian cancer is one of the most common and fatal malignant tumors threatening women’s health worldwide [1]. Ovarian cancer is ranked fourth and third globally in terms of incidence and mortality rates, respectively [1]. Serous ovarian cancer is the principal cause of death in patients with ovarian cancer. Continuous ovulation, low immunity, abnormal hormone fluctuation, and production of reactive oxygen species can lead to the occurrence of ovarian cancer [2]. Although the diagnosis and treatment of ovarian cancer have made unprecedented progress, the prognosis of patients with ovarian cancer remains dismal. The 5 years survival rate of ovarian cancer is lower than 50%, significantly lower than that of breast cancer (85%) [3]. Identifying new risk factors that regulate the occurrence, development, metastasis, and invasion of ovarian cancer will be helpful for early diagnosis and personalized treatment of patients with ovarian cancer. Therefore, establishing a novel prognostic signature of ovarian cancer and evaluating its potential physiological and pathological regulation mechanism is urgent to improve prognosis prediction and treatment strategy of ovarian cancer.

The tumor microenvironment (TME) is the determinant of cancer initiation and progression [4]. Various immune infiltrating cells in the TME interact with tumor cells, such as natural killer cells [5, 6], cytotoxic T cells [7, 8], and CD8+ T cells [9] and play an immune-promoting role and inhibit the occurrence and development of tumors. Immunosuppressive cells, such as fibroblasts [10, 11] and macrophages [12–14], play a tumor-promoting role. An analysis of 1731 patients with serous ovarian cancer showed that activated dendritic cells (DCs) were associated with a good prognosis [15, 16]. Moreover, it was previously demonstrated that the upregulation of immune related gene (IRG) expression in the TME was associated with the clinical outcome of ovarian cancer patients [17]. In addition, studies have preliminarily confirmed the predictive value of IRGs for serous ovarian cancer. Therefore, an immune-based prognostic signature still has intense application value in ovarian cancer.

Herein, we integrated multiple gene expression cohorts from different databases to develop and validate the prognostic signature of serous ovarian cancer based on IRGs. Differential expression analysis and univariate Cox proportional hazard regression analysis were performed on the training cohort in Gene Expression Omnibus (GEO) to screen IRGs with a prognostic value. These genes were further filtered through the LASSO-Cox regression model, and genes with the most prognostic value were selected for the construction of an immune related signature, which may be used as a prognostic indicator for patients with serous ovarian cancer.

## Materials and methods

### Patients

All tumor and normal tissue samples of patients with serous ovarian cancer were obtained from the Second Affiliated Hospital of Harbin Medical University. All patients were diagnosed with serous ovarian cancer by pathologists and clinicians. After surgical resection of the tumor focus, matched tumor and adjacent tissue specimens were collected from each patient. Finally, paired tissue specimens and clinical information from 26 patients were collected. This study was approved by the Clinical Research Ethics Committee of the Second Affiliated Hospital of Harbin Medical University.

### Data collection and processing

All data were obtained from an online public database. Gene expression profiles of serous ovarian cancer were downloaded from the GEO database. The inclusion criteria were as follows: (1) the sample size of the dataset is more than 20; (2) the dataset includes tumor tissue samples and normal tissue samples; (3) the sample source is the ovary. The exclusion criteria are as follows: (1) the dataset contains gene expression profiles of other cancers; (2) pathological examination confirms that the patient is not serous ovarian cancer; (3) the patient underwent chemotherapy or radiation therapy before sampling. The final data sets obtained included GSE26712, GSE18520, GSE137238, GSE36668, and GSE190688. In addition, the Count, transcript per million (TPM) gene expression profile, and clinical data of serous ovarian cancer were downloaded from The Cancer Genome Atlas (TCGA) database. The gene expression profile of normal ovarian tissues was downloaded from the Genotype-Tissue Expression (GTEx) database and compared with the gene expression profile of tumor tissues in TCGA.

The data obtained from the GEO database were corrected and normalized using the R package “limma” (version 3.50.1), and the data were transformed into log2. The “limma” provides an integrated solution for analyzing gene expression data. The functions of “limma” cover every major step of gene expression analysis, including data import, preprocessing, quality evaluation, normalization, differential expression analysis, and gene feature analysis. Data in TCGA and GTEx databases were annotated with gene ID using the GENCODE database, and TCGA and GTEx data sets were integrated into TCGA-GTEx cohorts.

GSE26712 and GSE18520 data sets were used as training cohorts and the rest as validation cohorts.

### Screening of differentially expressed genes

The R package “limma” was used to identify differentially expressed genes (DEGs) in the GEO training cohort. For all P-values, the false discovery rate (FDR) was used to correct the statistical significance of multiple tests. The threshold value of significant DEGs was |log2 fold change (FC)| > 1 and FDR < 0.05. To filter DEGs more scientifically, we calculated the standardized mean difference (SMD) for each gene. In the SMD analysis results, the threshold value for genes is |SMD| > 1.

### Deconvolution to calculate the proportion of immune cells

The CIBERSORT [18] algorithm was applied to quantify the proportion of 22 immune cells in the sample. CIBERSORT is an algorithm to estimate the relative proportion of 22 immune cells based on the gene expression profile. The “LM22.txt” file was downloaded from the CIBERSORTx website from (https://cibersort.stanford.edu/download.php) to run CIBERSORT. The “LM22.txt” is a “signature matrix”, containing 547 genes and 22 types of immune cells.

### Enrichment analysis and immune related gene set

The R package “clusterProfiler” (version 4.2.2) [19, 20] was used for Gene Ontology (GO) functional and Kyoto Encyclopedia of Genes and Genomes (KEGG) pathway enrichment analyses to predict the biological function of target genes [21, 22]. The “clusterProfiler” is an R package for Bioconductor that enables statistical analysis and visualization of functional clustering of gene sets or gene clusters. The functions of “clusterProfiler” mainly include functional enrichment analysis and visualization of enrichment results. At the same time, “clusterProfiler” can also be used for ID conversion of genes. In addition, the immune related gene set was downloaded from the Import database (https://www.immport.org/home). Finally, 456 immune related genes were obtained.

### Quantitative Real-Time real-time PCR (qRT-PCR)

Total RNA was extracted from 26 pairs of serous ovarian cancer and non-cancer tissues using RNA-easy Isolation Reagent (No. RC102-04, Vazyme, China). Then, the HiScript III first Strand Immunohistochemistry (IHC) was used for qRT-PCR. ChamQTM Universal SYBR® qPCR Master Mix (No. Q721-06, Vazyme, China), following the manufacturer’s instructions. Primer sequences are shown in Supplementary Table S1.

### Immunohistochemistry

Immunohistochemistry (IHC) analysis was performed according to the previous standard procedure. The anti-SST rabbit polyclonal antibody (Cat. No. ab187326; 1:100 dilution; Abcam, UK) and anti-DEFB1 rabbit polyclonal antibody (Cat. No. ab153562; 1:100 dilution; Abcam, UK) were added to the tissue section, and then incubated at 4 °C overnight. After washing with phosphate-buffered saline, the sample was further incubated with biotinylated secondary antibody (Cat. No. 111-024-005, No. 112-031-002; 1:1500 dilution; Jackson ImmunoResearch, USA) at room temperature for 30 min, and then exposed to diaminobenzidine at room temperature for 5 min. The entire experiment was repeated with phosphate-buffered saline instead of primary antibody to produce a negative staining control sample. More details are provided in Supplementary Table S2.

### Statistical analysis

We used the “surv_cutpoint” function in the R package “survminer” (version 0.4.9) to calculate the optimal cut-off and group patients. Kaplan–Meier survival curves for analysis of different patient groups were drawn based on the R package “survminer” (version 0.4.9) and “survival” (version 3.3-1), and the log-rank test was performed to determine the statistical significance of observed differences. The R package “pROC” (version 1.18.0) was used for receiver operating characteristic (ROC) curve analysis. The R package “rms” (version 6.3-0) was used to build the nomogram model and calibration curve. The “rms” is a collection of functions that assist with and streamline modeling. The “rms” works with almost any regression model, but it was especially written to work with binary or ordinal regression models, Cox regression, accelerated failure time models, ordinary linear models, the Buckley–James model, generalized least squares for serially or spatially correlated observations, generalized linear models, and quantile regression. The R package “ggplot2” (version 3.3.5) was used for drawing.

All statistical analyses were performed using the R programming language (version 4.2.0). Unless otherwise stated, all statistical tests were two-sides tests, and P < 0.05 was considered statistically significant.

## Results

### Differential expression analysis at the transcriptome level

**Fig. 1:**
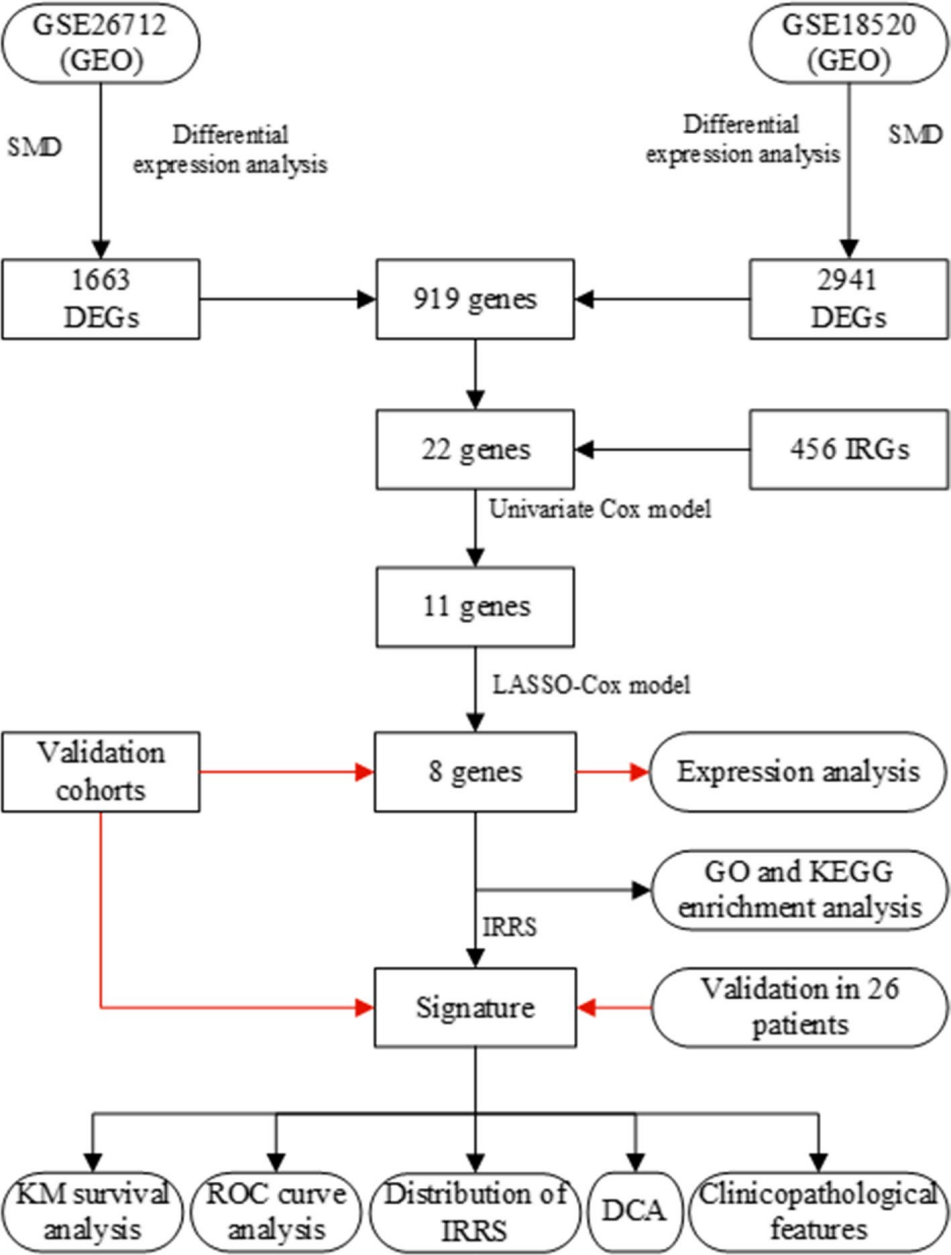
This is the flow chart

**Fig. 2:**
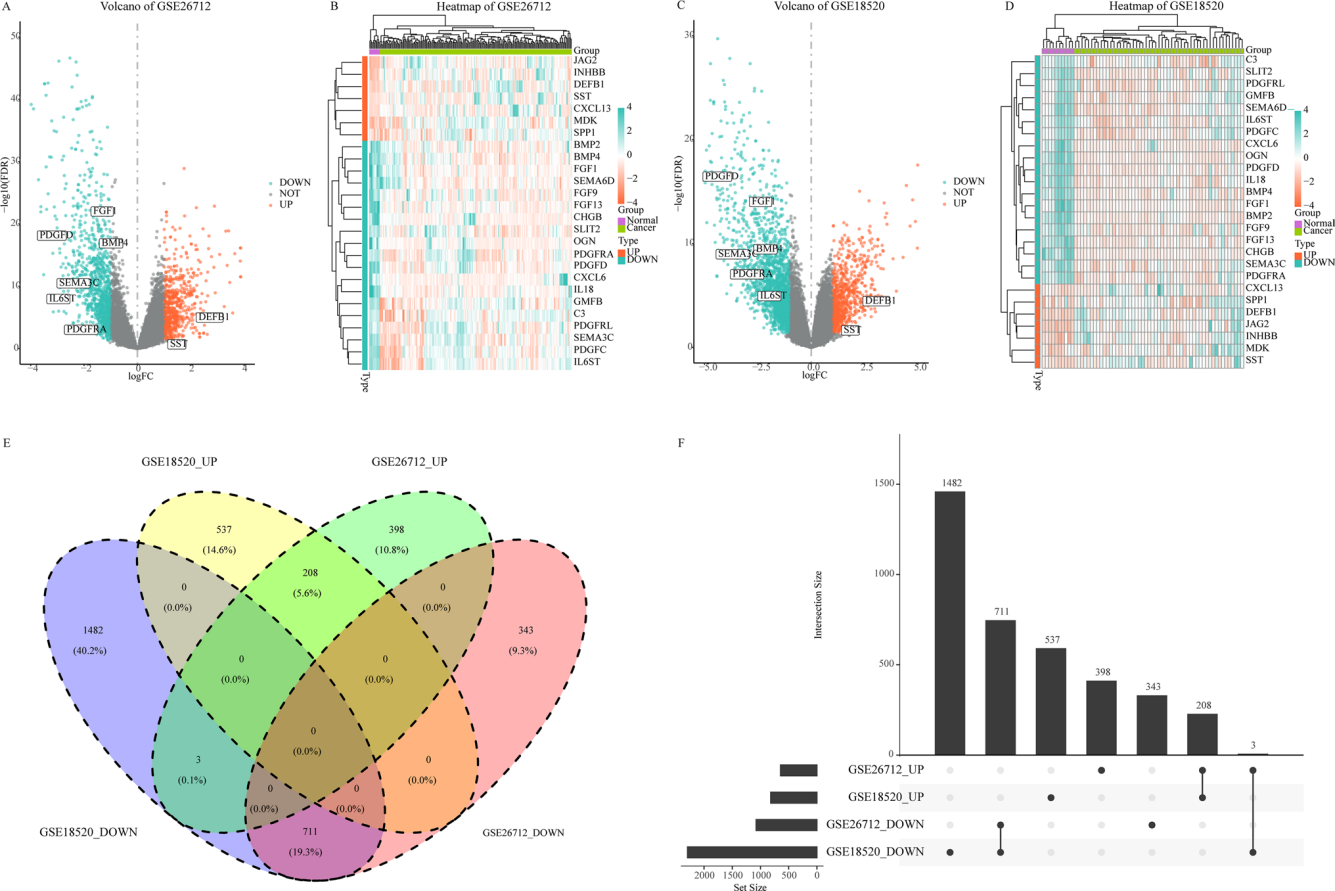
Identify differentially expressed genes. **A** Volcano map of differentially expressed genes in data set GSE26712, with significantly up-regulated genes in red and significantly down-regulated genes in blue; **B** heatmaps of 50 significantly differentially expressed genes in dataset GSE26712; **C** volcano map of differentially expressed genes in dataset GSE18520; **D** heatmaps of 50 significantly differentially expressed genes in dataset GSE18520; **E** Venn diagram shows the intersection of differentially expressed genes in two data sets in the training cohort; **F** upset maps show the intersection of differentially expressed genes in two data sets in the training cohort

The schematic diagram of this study is shown in Fig. 1. We used the R software package “limma” to perform differential expression analysis and calculate SMD on the gene expression profiles of the GSE26712 and GSE18520 datasets in the training cohort. And take the overlapping between the “limma” package and SMD analysis results. A total of 1663 DEGs were identified in the GSE26712 data set, of which 1054 were significantly downregulated genes and 609 were significantly upregulated genes (Fig. 2a). By mapping some DEGs, it was found that the expression level of these genes was significantly different between normal and tumor samples (Fig. 2b). A total of 2941 DEGs were identified in the GSE18520 in the data set, of which, 2196 were significantly downregulated genes and 745 were significantly upregulated genes (Fig. 2c). The expression level of some DEGs in normal and tumor samples is shown in Fig. 2d.

DEGs in GSE26712 and GSE18520 data sets were crossed to further identify significant overlaps between genes in the two data sets. Finally, 208 upregulated and 711 downregulated genes were identified (Fig. 2e, f).

### Immune related genes with prognostic significance

**Fig. 3:**
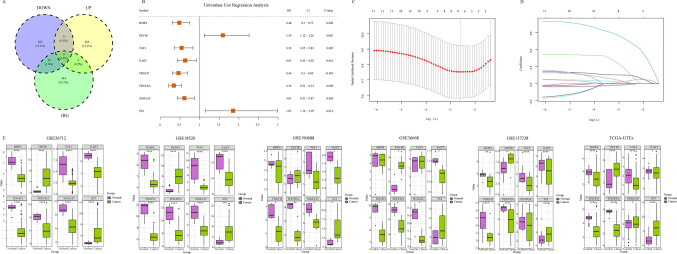
Filtering of immune related genes. **A** The intersection of up-regulated or down-regulated genes and immune related genes at the same time; **B** the forest map shows the risk ratio of some of the 13 genes in the data set GSE26712; **C** the LASSO regression model shows partial likelihood deviation in the tenfold cross validation, and draws a vertical dotted line at the optimal value using the minimum value criterion and 1-SE criterion; **D** distribution of LASSO coefficients of 13 genes; **E** the expression difference of 8 genes between normal and tumor tissues in the training and validation cohort

Next, these 919 DEGs and were crossed with 456 IRGs to screen out immune regulated DEGs, and 3 upregulated and 19 downregulated immune regulated DEGs were obtained (Fig. 3a).

To further confirm whether these IRGs were associated with the prognosis of serous ovarian cancer, we conducted a univariate Cox proportional hazard regression analysis. The results showed that the hazard ratios (HR) of 11 IRGs were statistically significant (Fig. 3b). The HR of most genes was less than 1, indicating that these IRGs may be potential prognostic factors and could inhibit the occurrence and development of serous ovarian cancer. Subsequently, the LASSO Cox regression model was used to screen IRGs that were highly associated with patient prognosis. In this model, lambda.1se was 0.2531608 (Fig. 3c, d). The results showed that the coefficients of eight IRGs were not equal to 0. The eight IRGs, including IL6ST, DEFB1, BMP4, SEMA3C, SST, DGFRA, PDGFD, and FGF1, were selected for further analysis.

All genes were lowly expressed in tumor samples, except for DEFB1 and SST (Fig. 2e). To further verify the expression of the eight genes in tumors, several validation data sets were analyzed. As expected, all genes were lowly expressed in tumor tissues of multiple validation data sets, except for DEFB1 and SST, which were highly expressed in tumor tissues (Fig. 3e).

### Enrichment analysis of the IRGs

**Fig. 4:**
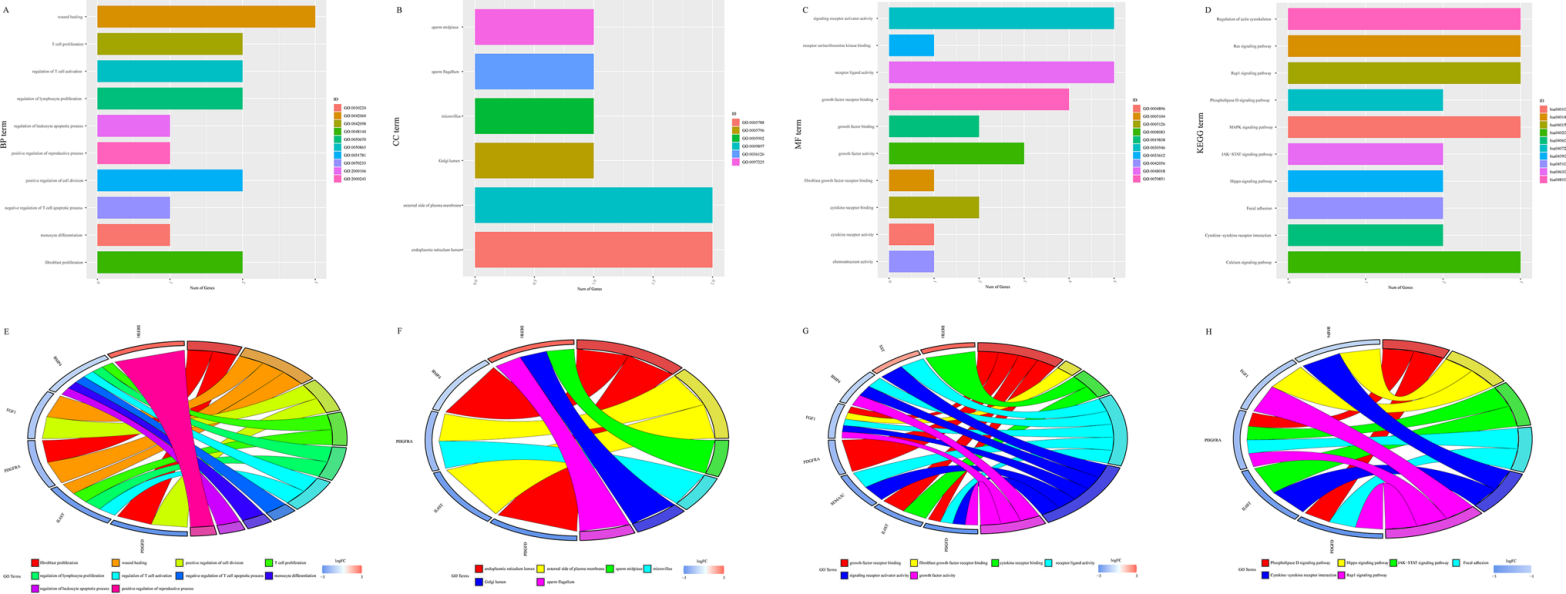
Enrichment analysis of immune related genes. **A**–**D** Respectively, are the histogram of the enrichment analysis results of 8 immune related genes at BP, CC, MF and KEGG levels; **E**–**H** respectively, are the circles of the results of the enrichment analysis of 8 immune related genes at BP, CC, MF and KEGG levels. The left half of the circle is the gene name, which is sorted by logFC from bottom to top. The right half of the circle is the name of term. The line between the gene and term indicates that the gene exists on the term

After filtering genes through the LASSO Cox model, only 8 of 11 genes had a potential biological regulation relationship with the progression of serous ovarian cancer. To further explore the biological functions of IRGs, we performed GO functional and KEGG pathway enrichment analyses on the eight IRGs. At the biological process (BP) level, it was found that these genes have a significantly enriched in immune related functions and cell proliferation, such as fibroblast promotion, T cell promotion, regulation of lymphocyte promotion, regulation of T cell activation, and positive regulation of cell division (Fig. 4a). At the cell composition (CC) level, these genes were found to participate in endoplasmic reticulum lumen, external side of the plasma membrane, Golgi lumen and microvillus (Fig. 4b). Moreover, enrichment analysis revealed that the genes were related to tumor inflammation responses, such as receiver light activity, signaling receiver activator activity, growth factor activity, and chemoattractant activity at the molecular function (MF) level (Fig. 4c). In the KEGG enrichment pathway analysis, it was found that genes were enriched in carcinogenic pathways related to serous ovarian cancer, such as the Hippo signaling pathway, the Janus kinase-signal transducers and activators of transcription (JAK-STAT) signaling pathway, Focal induction, the Ras signaling pathway, and the Phospholipase D signaling pathway (Fig. 4d). In addition, the relationship between the above terms and the eight DEGs was demonstrated (Fig. 4e–h).

### Prognostic value of immune related signature in serous ovarian cancer

**Fig. 5:**
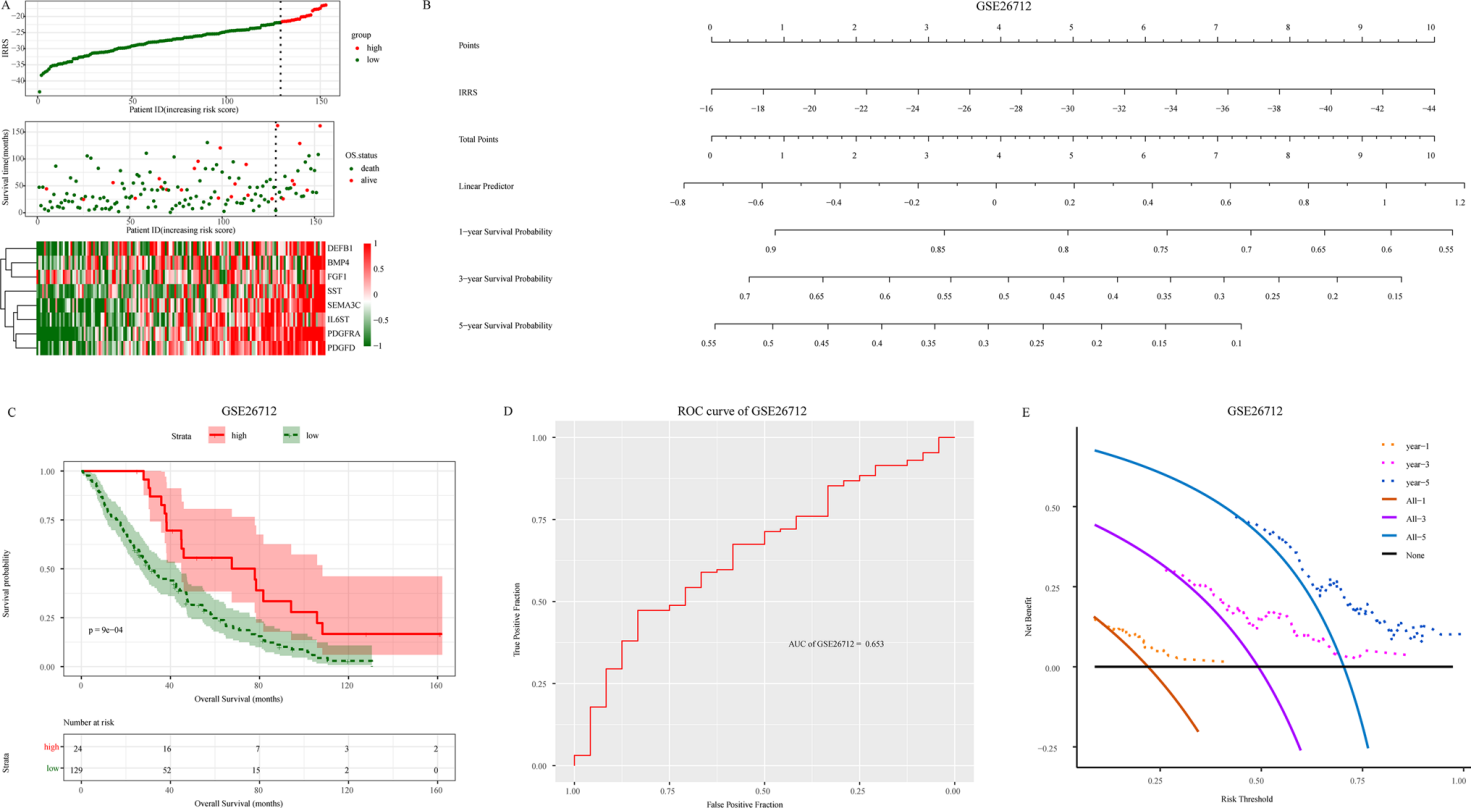
The training cohort GSE26712 constructs immune related prognostic signatures. **A** Risk factor correlation diagram, above figure: the risk score of each patient is ranked from small to large. The vertical dotted line represents the best cutoff value of the risk score, from which the low-risk group (green) and high-risk group (red) can be distinguished; Middle figure: the relationship between patients and survival time according to the risk score. The green dot represents the dead patients, and the red represents the living patients; The following figure shows the expression pattern of 8 genes in patients; **B** calculate the risk score of the signature and build the nomogram model; **C** according to the best cutoff value of risk score, patients were divided into high score group and low score group, and survival analysis was performed; **D** ROC curve analysis of the signature; **E** analysis of the 1, 3 and 5 years decision curve of the signature

We constructed an immune- related prognostic signature using eight genes and calculated an immune related risk score (IRRS) for each patient based on the formula $\sum\nolimits_{i}^{8}log2$ (HR_i_)*expr_i_ (IRRS, i represents eight genes). The best cut-off value based on the IRRS of each patient was calculated and patients were divided into a high-score group and a low score group. Figure 5a shows the distribution of the IRRS, overall survival time, survival status, and the expression mode of the eight genes. The results showed that the death rate of patients in the low score group was significantly higher than that in the high-score group.

Then, a nomogram model of the immune related signature was constructed based on the IRRS (Fig. 5b). As anticipated, the IRRS of the immune related signature was positively correlated with the survival probability of patients. Kaplan–Meier survival curve analysis showed that patients in the high-score group had a longer overall survival time, suggesting that higher IRRS may be a potential inhibitor of serous ovarian cancer (Fig. 5c). To determine the accuracy of the immune related signature in predicting the prognosis of patients, we performed a ROC curve analysis. The results revealed that the area under the ROC curve (AUC) was 0.65, indicating that immune related signature had a good predictive effect on the prognosis of patients (AUC = 0.65, Fig. 5d). Meanwhile, decision curve analysis showed that the model yielded a high clinical net benefit in 1, 3, and 5 years, and thus the nomogram model can well predict the survival probability of 1, 3 and 5 years (Fig. 5e). Overall, these results indicate that the nomogram model of immune related signature has strong identification and calibration capabilities.

### Validation of the prognostic value of the immune related signature

**Fig. 6:**
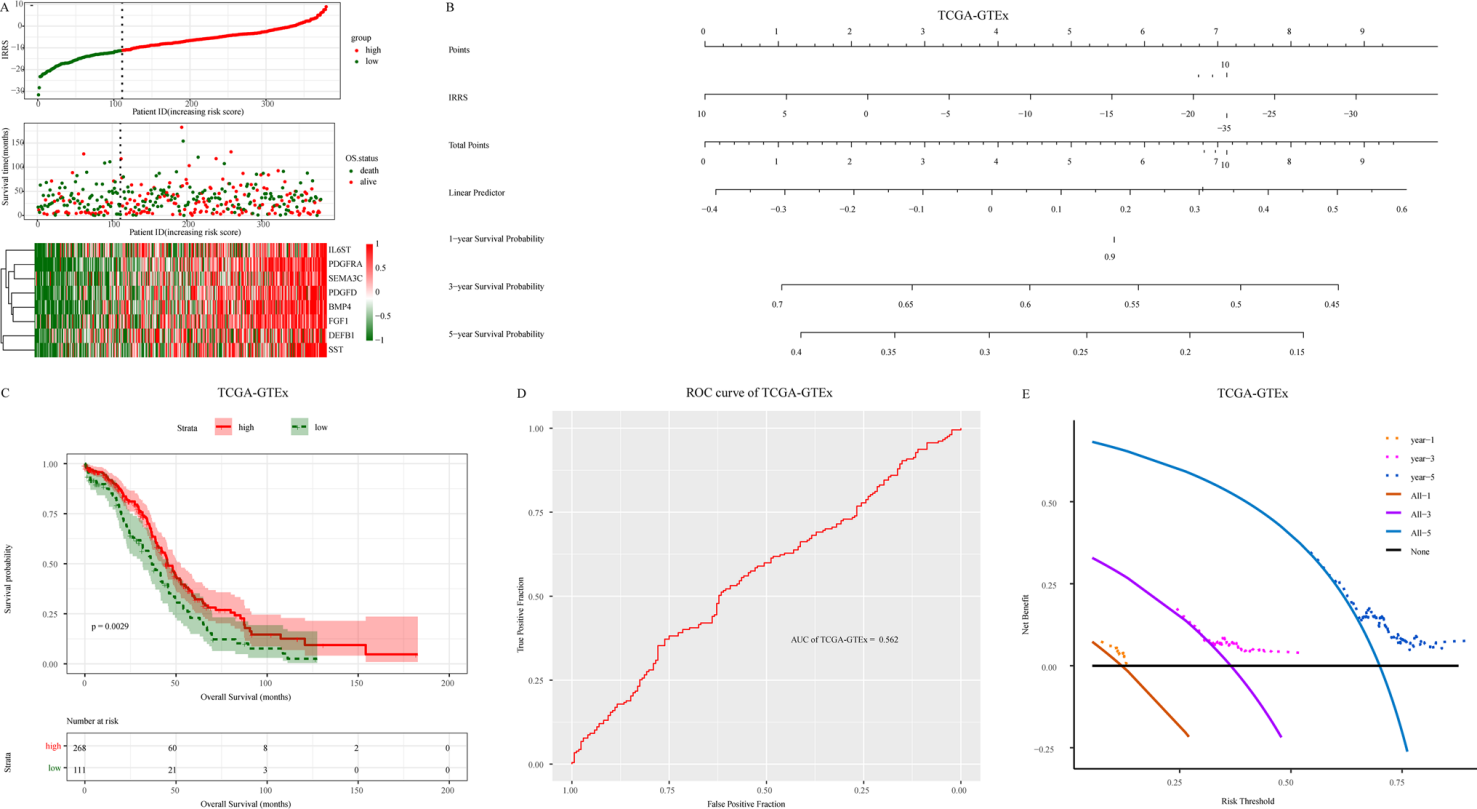
Validation of the testing cohort TCGA-GTEx. **A** Risk factor correlation diagram, above figure: the risk score of each patient is ranked from small to large. The vertical dotted line represents the best cutoff value of the risk score, from which the low-risk group (green) and high-risk group (red) can be distinguished; Middle figure: the relationship between patients and survival time according to the risk score. The green dot represents the dead patients, and the red represents the living patients; The following figure shows the expression pattern of 8 genes in patients; **B** calculate the risk score of the signature and build the nomogram model; **C** according to the best cutoff value of risk score, patients were divided into high score group and low score group, and survival analysis was performed; **D** ROC curve analysis of the signature; **E** analysis of the 1, 3 and 5 years decision curve of the signature

To validate the accuracy of immune related signature prediction efficiency, we performed a similar validation analysis using the gene expression profile of serous ovarian cancer in TCGA. Although most patients showed higher IRRS in the validation data set, their death rate was still lower than that of the lower group (Fig. 6a). The nomogram model (Fig. 6b) and Kaplan Meier survival curve analysis (Fig. 6c) of the validation dataset showed similar trends. Although the results of ROC curve analysis (AUC = 0.56, Fig. 6d) and decision curve analysis (Fig. 6e) suggested that the prediction efficiency and clinical benefits of immune related signature were low in the validation data set, patients still benefited from the nomogram model of immune related signature. In addition, to ensure the robustness of the research results, GSE30161 and GSE63885 data sets were used to reverify the experimental results. The results confirmed the robustness of the prognostic signature (Supplementary Figure 1).

The results of the validation data set further suggest that the immune related signature has high accuracy in predicting the prognosis of patients with serous ovarian cancer, and may benefit patients in clinical decision-making.

### Expression of signature-related genes in serous ovarian cancer

**Fig. 7:**
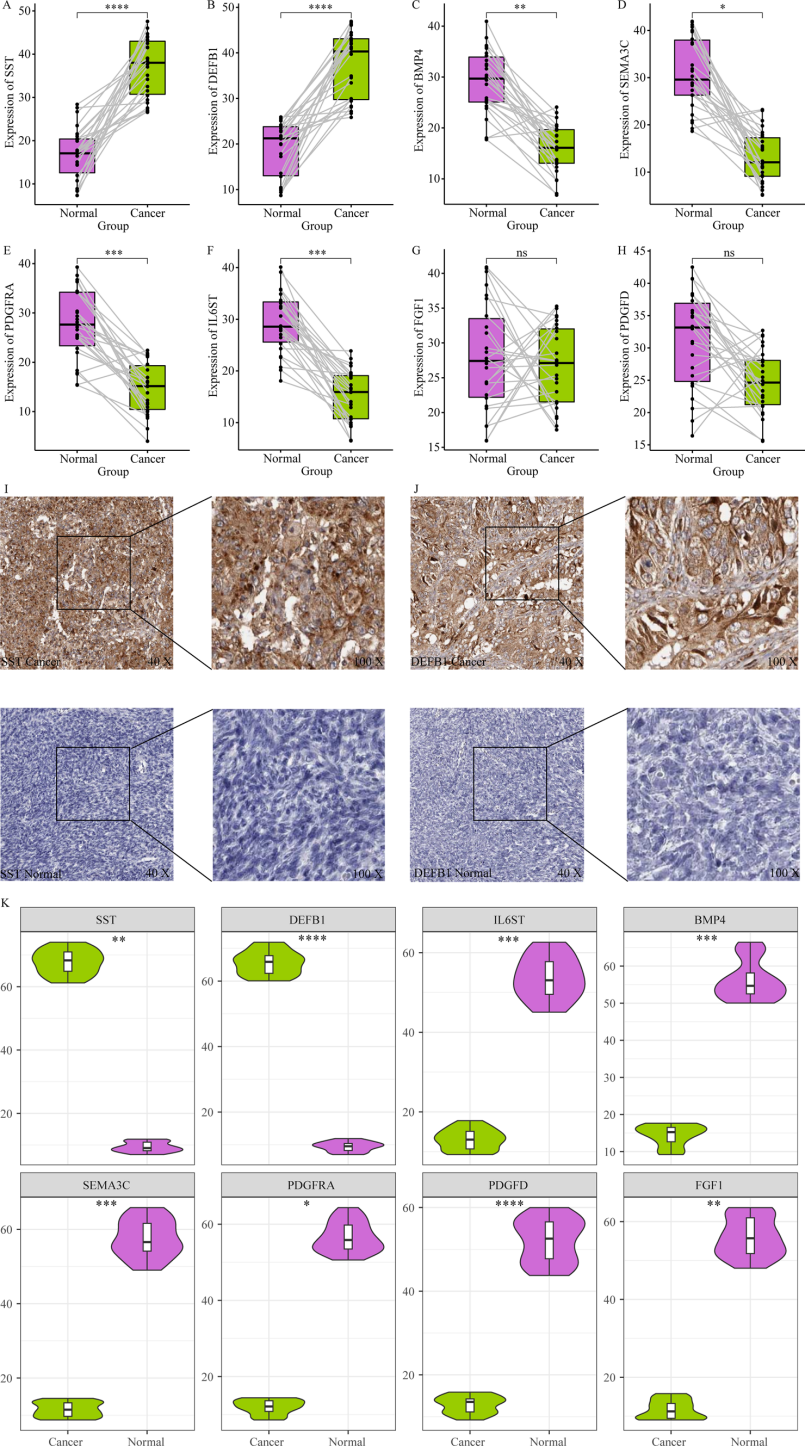
The expression of 8 genes in human tissue specimens. **A**–**H** QRT-PCR was employed to test the expression level of 8 genes in serous ovarian cancer patients. **I**, **J** Immunohistochemistry was employed to test the protein level of SST and DEFB1 in serous ovarian cancer patients. **K** the statistical results of IHC images in 8 genes by ImageJ tool. *p-value < 0.05, **p-value < 0.01, ***p-value < 0.001, ****p-value < 0.0001, ns is not significant

Furthermore, to further verify the accuracy of the signature, we collected clinical information and tissue samples from 26 patients. Patients were grouped based on the location of the tumor, and the baseline information is shown in Table 1. The expression of eight signature-related genes in the clinical samples of patients with serous ovarian cancer was examined by qRT-PCR analysis. The results showed that the expression of SST and DEFB1 was higher in serous ovarian cancer tissues (Fig. 7a, b), while the expression of the other six genes was lower in tumor tissues (Fig. 7c–h). IHC analysis of the eight genes also showed a similar trend (Fig. 7i–k, Supplementary Figure 2).

**Table 1:** The baseline information of serous ovarian cancer by groups of tumor location

	Bilateral (N = 10)	Left (N = 6)	Right (N = 10)	p.overall
Age	53.6 (11.9)	64.2 (11.5)	60.3 (13.2)	0.238
T stage				0.375
1	0 (0.00%)	1 (16.7%)	0 (0.00%)	
2	7 (70.0%)	2 (33.3%)	5 (50.0%)	
3	3 (30.0%)	3 (50.0%)	5 (50.0%)	
N stage				0.686
0	7 (70.0%)	4 (66.7%)	5 (50.0%)	
1	3 (30.0%)	2 (33.3%)	5 (50.0%)	
M stage				1.000
0	9 (90.0%)	5 (83.3%)	9 (90.0%)	
1	1 (10.0%)	1 (16.7%)	1 (10.0%)	
Stage				0.695
I	0 (0.00%)	1 (16.7%)	0 (0.00%)	
II	5 (50.0%)	1 (16.7%)	5 (50.0%)	
III	4 (40.0%)	3 (50.0%)	4 (40.0%)	
IV	1 (10.0%)	1 (16.7%)	1 (10.0%)	
Grade				< 0.001
G1	2 (20.0%)	0 (0.00%)	4 (40.0%)	
G2	8 (80.0%)	3 (50.0%)	0 (0.00%)	
G3	0 (0.00%)	3 (50.0%)	6 (60.0%)

### Immune related signature affects the functional status and tumor invasion abundance of DCs

**Fig. 8:**
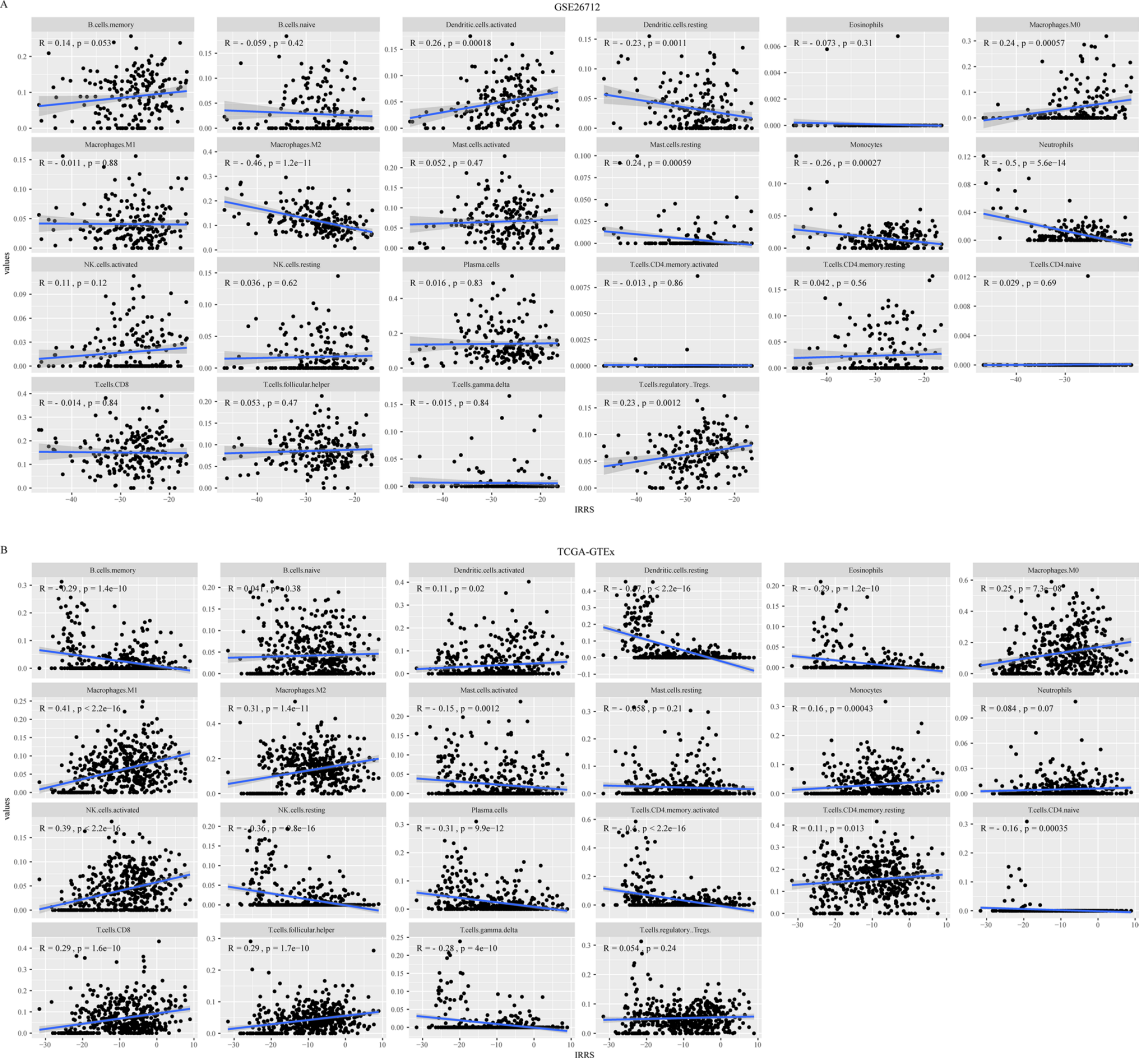
Abundance analysis of immune infiltrating cells. **A**, **B** Represent the correlation analysis between the risk score in the data set GSE26712 and TCGA-GTEx and the abundance of 22 immune cells in CIBERSORT

Since genes in the immune related signature are related to immune function, we analyzed the correlation between the signature and the abundance of the 22 types of infiltrating immune cells. The infiltration abundance of most immune cells showed an inconsistent trend in different data sets. However, activated and resting DCs showed a corresponding trend in the two data sets (Fig. 8a, b). Interestingly, the IRRS of the signature was positively correlated with the infiltration abundance of activated DCs but negatively correlated with the infiltration abundance of resting DCs. This suggests that the tumor inhibition effect of the immune related signature may be related to the functional state of DCs, and may exert the tumor inhibition effect by affecting the functional state and infiltrating the abundance of DCs.

## Discussion

The clinical results of patients with serous ovarian cancer are patient specific, and their survival time ranges from 5 months to over 10 years [23]. At present, the main treatment strategy for serous ovarian cancer include radical surgery and chemotherapy, which significantly prolong the recurrence interval; however, the overall survival time of patients remains poor [24]. Hence, a wide range of prognostic signatures is needed to accurately identify patients with refractory diseases and poor survival rates. Although some recognized clinical phenotypes, such as tumor staging and grading, have been widely used to guide patients to receive adjuvant chemotherapy after surgery, this cannot fully distinguish patients with an increased risk of tumor progression [25]. Therefore, considerable attention should be paid to the molecular mechanism underlying the occurrence and development of serous ovarian cancer to reveal the main factors related to clinical results.

In the present study, we used multiple public serous ovarian cancer data sets to develop a prognostic signature based on IRGs. The establishment of the immune related signature was achieved through a multi-step bioinformatics analysis method. First, we analyzed the differential expression of the training cohort data set and identified genes that were simultaneously up-regulated or down-regulated in the training dataset. Meanwhile, we crossed the genes that were upregulated or downregulated with IRGs to screen out immune related DEGs. Then, the univariate Cox proportional hazard regression model was used to analyze the impact of IRGs on the prognosis of serous ovarian cancer, and genes with the strongest correlation with prognosis were identified through the LASSO Cox regression model. Finally, eight IRGs were identified and an immune related prognosis signature was constructed based on eight IRGs. Of the eight IRGs, only DEFB1 [26] and SST [27] were highly expressed in tumor tissues, while the other six genes were lowly expressed. This finding is consistent with results reported in previous studies [28, 29].

We performed a systematic prognostic correlation analysis of immune related signature by calculating their IRRS in each patient. Our data showed that the death rate of patients in the low-score group was significantly higher than that in the high-score group. The nomogram model also demonstrated that the IRRS of the signature was negatively correlated with the survival probability of ovarian cancer. Kaplan–Meier survival curve analysis further verified this result. A ROC curve analysis was conducted to judge the prediction efficiency of the signature. Both the training and validation data sets showed a high AUC, suggesting that the signature had a strong prediction efficiency.

To further analyze the biological function of the gene signature in tumors, we conducted function and pathway enrichment analyses. Interestingly, these IRGs were widely involved in immune related functions, such as fibroblast promotion, T cell promotion, regulation of lymphocyte promotion, and regulation of T cell activation. Meanwhile, these genes were also involved in some carcinogenic related pathways, including the Hippo signaling pathway [30], the JAK-STAT signaling pathway [30, 31], Focal adhesion, the Ras signaling pathway [32], and the Phospholipase D signaling pathway. These results suggested that these genes may affect the occurrence and development of tumors by mediating immune related functions.

Deconvolution analysis of the training and validation data sets showed that the IRRS of this signature was positively correlated with the infiltration abundance of activated DCs, but negatively correlated with the infiltration abundance of rest DCs. This is consistent with findings from previous reports. Activated DCs improve the prognosis of patients with serous ovarian cancer [15]. Our data also suggested that the IRRS of this signature was positively associated with the survival time of patients with serous ovarian cancer.

Nonetheless, this study has several limitations. Firstly, this retrospective study is based on previously published data and therefore requires more real-time clinical data for prospective analysis and verification. Secondly, we did not carry out experimental verification on the theoretical analysis results due to the epidemic situation. Future studies should be conducted with more basic experiment methods, such as Western blotting, co-immunoprecipitation and other molecular biological methods to validate our findings and clarify the potential molecular mechanism of serous ovarian cancer.

## Conclusions

In summary, a prognostic signature based on eight IRGs, including IL6ST, DEFB1, BMP4, SEMA3C, SST, DGFRA, PDGFD and FGF1, was successfully constructed via systematic bioinformatics analysis. The IRRS of the signature was calculated and used for further analysis. The IRRS suggested that this signature could better predict the prognosis of patients with serous ovarian cancer, and inhibit the occurrence and development of tumors. Further analysis confirmed that the tumor inhibitory effect of this signature may be linked to the infiltration abundance of activated DCs.

## Supplementary Information

Below is the link to the electronic supplementary material.

Supplementary file 1 (DOCX 866 KB)

Supplementary file 2 (XLSX 10 KB)

## Data Availability

The datasets analyzed during the current study are available in the Gene Expression Omnibus (GEO) and The Cancer Genome Atlas (TCGA) repository. The web link of GEO is https://www.ncbi.nlm.nih.gov/geo/, datasets in GEO includes GSE26712, GSE18520, GSE137238, GSE36668 and GSE190688. The web link of TCGA is https://portal.gdc.cancer.gov/repository.
