# Supplementary material for: An immune related signature inhibits the occurrence and development of serous ovarian cancer by affecting the abundance of dendritic cells
Source: Discov Oncol. 2023 Jun 15;14:101. doi: 10.1007/s12672-023-00717-z (PMC10271965; doi:10.1007/s12672-023-00717-z)
Supplement: Supplementary file 1 — (DOCX 866 KB) [file 12672_2023_717_MOESM1_ESM.docx]

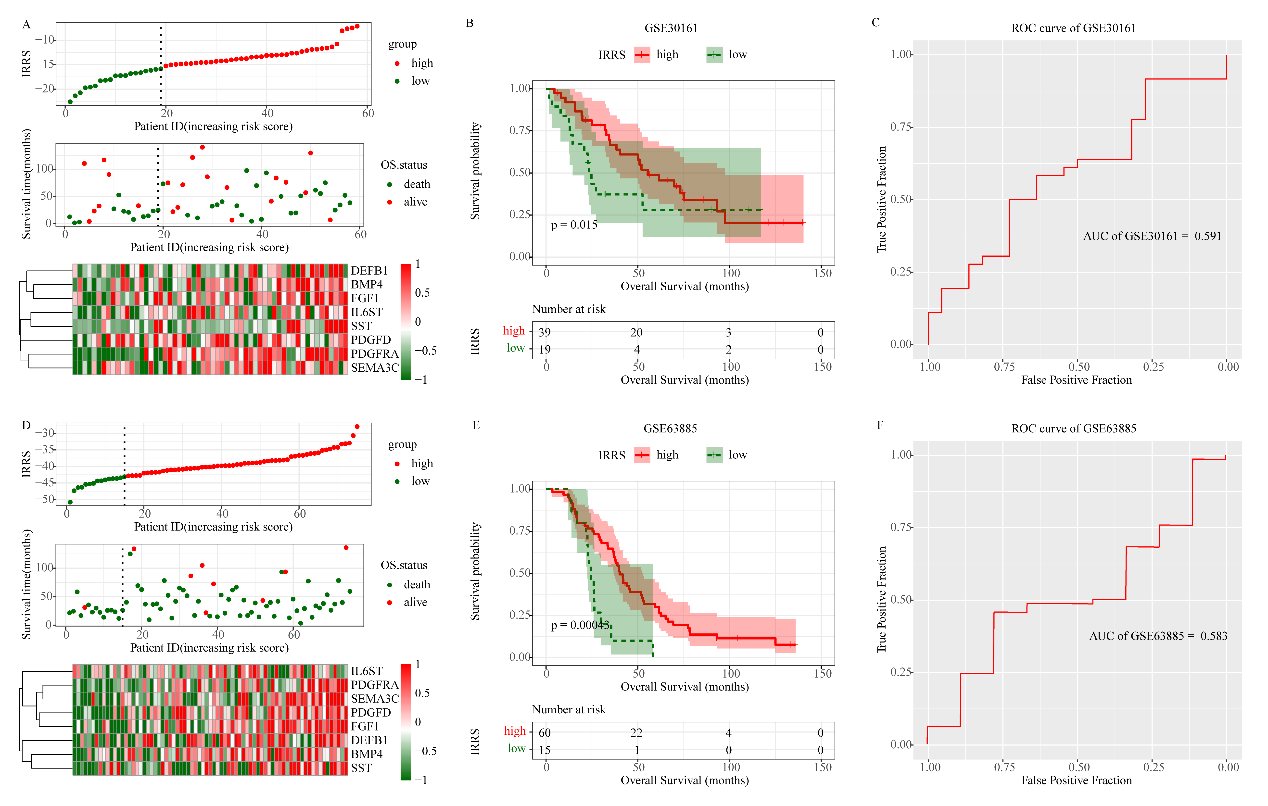


Supplementary Figure 1. Validation of the results. A-C, GSE30161 cohort. D-F, GSE63885 cohort.


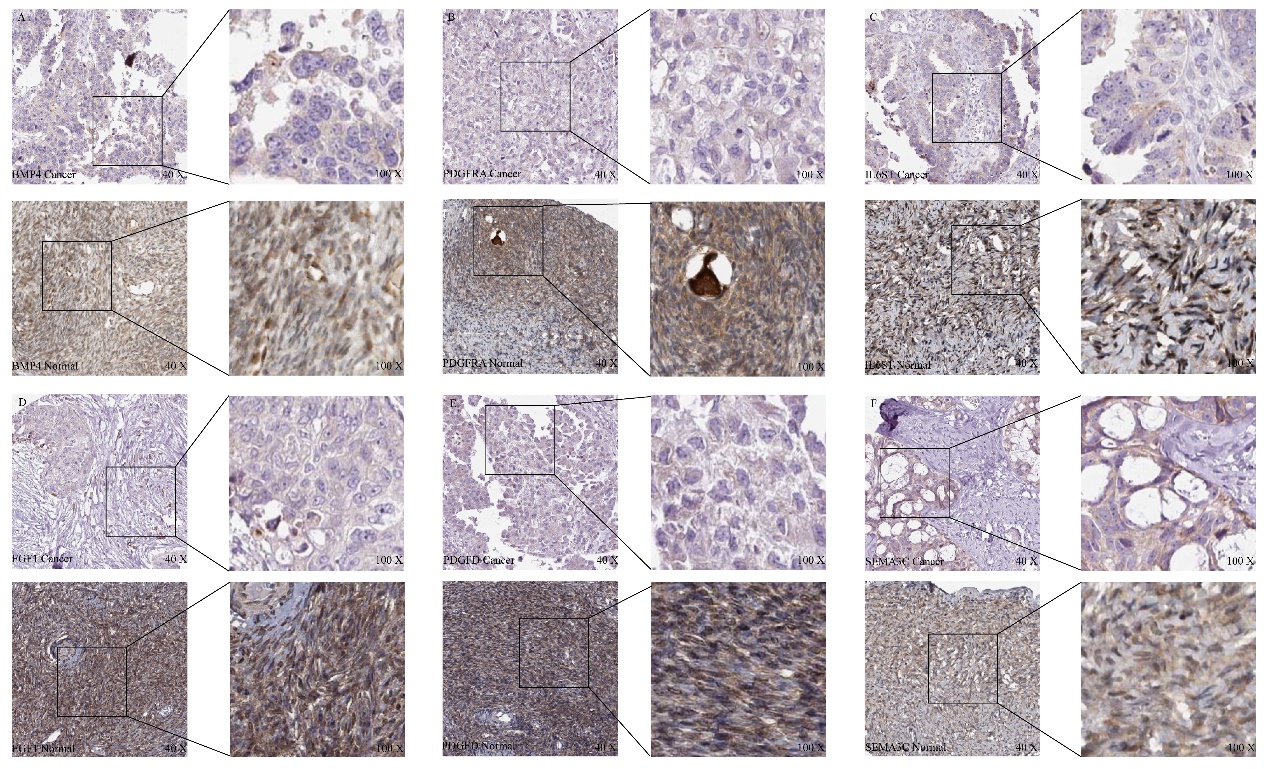


Supplementary Figure 2. Immunohistochemistry analyses of 6 genes in human tissue specimens. A-F, representing IL6ST, DEFB1, BMP4, SEMA3C, SST, PDGFRA, PDGFD and FGF1, respectively.
